# LANTERN-XGB: An Interpretable Multi-Modal Machine Learning for Improving Clinical Decision-Making in Lung Cancer

**DOI:** 10.3390/ijms27073128

**Published:** 2026-03-30

**Authors:** Davide Dalfovo, Carolina Sassorossi, Elisa De Paolis, Annalisa Campanella, Dania Nachira, Leonardo Petracca Ciavarella, Luca Boldrini, Esther G. C. Troost, Róza Ádány, Núria Farré, Ece Öztürk, Angelo Minucci, Rocco Trisolini, Emilio Bria, Steffen Löck, Stefano Margaritora, Filippo Lococo

**Affiliations:** 1 OncoRay—National Center for Radiation Research in Oncology, Faculty of Medicine, University Hospital Carl Gustav Carus, TUD Dresden University of Technology, Helmholtz-Zentrum Dresden-Rossendorf, 01067 Dresden, Germany; davide.dalfovo@tu-dresden.de (D.D.);; 2 Department of Radiotherapy and Radiation Oncology, Faculty of Medicine, University Hospital Carl Gustav Carus, TUD Dresden University of Technology, 01067 Dresden, Germany; 3 Unit of Thoracic Surgery, Catholic University of the Sacred Heart, 00168 Rome, Italydania.nachira@policlinicogemelli.it (D.N.); stefano.margaritora@policlinicogemelli.it (S.M.); 4 Thoracic Surgery Unit, A. Gemelli University Hospital Foundation IRCCS, 00168 Rome, Italy; 5 Departmental Unit of Molecular and Genomic Diagnostics, Genomics Core Facility, Gemelli Science and Technology Park (G-STeP), A. Gemelli University Hospital Foundation IRCCS, 00168 Rome, Italy; elisa.depaolis@policlinicogemelli.it (E.D.P.);; 6 Clinical Chemistry, Biochemistry and Molecular Biology Operations (UOC), A. Gemelli University Hospital Foundation IRCCS, 00168 Rome, Italy; 7 Advanced Radiotherapy Center, A. Gemelli University Hospital Foundation IRCCS, 00168 Rome, Italy; 8 Institute of Radiooncology—OncoRay, Helmholtz-Zentrum Dresden-Rossendorf, 01067 Dresden, Germany; 9 ELKH-DE Public Health Research Group, Department of Public Health and Epidemiology, Faculty of Medicine, University of Debrecen, 4001 Debrecen, Hungary; 10 Institut de Recerca de L’Hospital de la Santa Creu i Sant Pau (IR-HSCSP), 08001 Barcelona, Spain; 11 School of Medicine, Turkey and Koç University Research Center for Translational Medicine (KUTTAM), Koç University, Istanbul 34450, Turkey; 12 Interventional Pulmonology Unit, A. Gemelli University Hospital Foundation IRCCS, 00168 Rome, Italy; 13 Medical Oncology, A. Gemelli University Hospital Foundation IRCCS, Largo a. Gemelli 8, 00168 Rome, Italy

**Keywords:** multi-modal integration, artificial intelligence, precision oncology, lung cancer, radiogenomics

## Abstract

Non-small cell lung cancer (NSCLC) remains the leading cause of cancer-related mortality globally. While multi-modal artificial intelligence (AI) models offer significant predictive potential, their translation into routine clinical practice is delayed by the “black box” nature of complex algorithms and the fragmentation of heterogeneous data. We present LANTERN-XGB, a hierarchical machine learning workflow designed to bridge this gap by generating interpretable “digital human avatars” for precision oncology. The methodology employs a multi-stage scalable tree boosting system (XGBoost) architecture utilizing shapley additive explanations (SHAP) for rigorous hierarchical feature selection, missing value management, and patient-specific decision support. The workflow was developed and benchmarked using a retrospective cohort of 437 patients with clinical N0 NSCLC, followed by validation on a prospective dataset (n = 100) and an independent external dataset (*n* = 100). The pipeline integrates diverse data modalities to predict occult lymph node metastasis (OLM). LANTERN-XGB identified a robust consensus signature driven by non-linear interactions among CT textural fragmentation, PET metabolic heterogeneity, tumor density distribution, and systemic clinical modulators. Exploratory transcriptomic pathway analysis (GSVA) revealed that high-risk predictions strongly correlate with systemic molecular dysregulation, such as the enrichment of immune-inflammatory signaling and metabolic stress pathways. The model achieved robust discrimination in external validation (AUC ≈ 0.77), performing comparably to state-of-the-art nomogram benchmarks. Crucially, the LANTERN-XGB framework demonstrated superior utility in handling diagnostic ambiguity; local force plots allowed for the correct reclassification of “borderline” prediction by visualizing feature interactions that standard linear models fail to capture. LANTERN-XGB provides a validated, open-source framework that successfully balances predictive power with clinical transparency. By empowering clinicians to visualize and verify the logic behind AI predictions, this workflow offers a pragmatic path for integrating reliable multi-modal avatars into daily medical decision-making.

## 1. Introduction

Lung cancer represents the most substantial challenge in global oncology, retaining its position as the leading cause of cancer-related mortality worldwide. Recent global estimates indicate that lung cancer accounts for approximately 2.5 million new diagnoses each year and is responsible for nearly 18% of all cancer-related deaths, exceeding the combined mortality burden of breast, colorectal, and prostate cancers [1]. Non-small cell lung cancer (NSCLC) constitutes approximately 85% of all lung cancer cases. Despite major therapeutic advances, including the development of third-generation tyrosine kinase inhibitors and immune checkpoint inhibitors, survival outcomes for patients with advanced-stage NSCLC remain poor, with 5-year survival rates showing only modest improvement [2]. This persistent mortality burden reflects, in part, the profound biological heterogeneity of NSCLC and the inherent limitations of conventional TNM staging systems, which inadequately capture the molecular complexity required to inform optimal therapeutic decision-making.

In response to these challenges, computational biology and artificial intelligence (AI) have emerged as critical tools for advancing NSCLC diagnosis, staging, and prognostication. In particular, machine learning (ML) and deep learning (DL) approaches applied to high-dimensional imaging and molecular datasets have demonstrated considerable potential to improve traditional clinical workflows [3]. Recent investigations have shown that AI models trained on thoracic computed tomography images can achieve diagnostic performances exceeding 80% for the prediction of occult N2 lymph node metastasis, significantly outperforming conventional statistical approaches and standard clinical assessment [4]. These findings underscore the capacity of AI-driven imaging analysis to enhance staging precision. This potential is further corroborated by large-scale validation studies; for instance, a deep learning survival model utilizing combinatorial TNM data across 17,000 patients demonstrated a concordance index (C-index) of 0.83, proving significantly more stable and accurate than the standard TNM staging system (C-index 0.65) in predicting patient outcomes [5,6]. Beyond traditional radiology, AI is also reshaping histopathological assessment, with deep convolutional neural networks now capable of predicting specific driver mutations, such as *EGFR*, directly from standard H&E-stained slides with performances reaching 97%, offering a rapid, non-invasive alternative to molecular sequencing [7].

Beyond single-modality assessments, integrative multi-modal strategies are increasingly recognized as superior for prognostic stratification in NSCLC. Recent investigations have demonstrated that combined analyses of genomic, transcriptomic, and proteomic features can identify robust prognostic signatures that stratify patient risk more effectively than individual biomarkers alone [8]. A notable example of this evolution is the integration of broad clinical data with novel biological layers; recent work utilizing a scalable tree boosting system (XGBoost) on perioperative data and blood biomarkers outperformed pathological staging in predicting disease-free and overall survival [9]. Furthermore, in the complex landscape of immunotherapy, plasma proteomics models like “PROphet” have emerged, demonstrating the ability to predict clinical benefit from PD-1/PD-L1 inhibitors with greater precision than PD-L1 expression alone, thereby preventing unnecessary toxicity and optimizing combination strategies [10]. These advancements collectively signal a paradigm shift toward holistic, AI-driven “digital avatars” that capture the full heterogeneity of the patient’s disease.

Despite these methodological advances, a substantial translational gap persists between algorithmic innovation and clinical implementation. Although numerous high-performing predictive models have been reported, their adoption in routine clinical practice remains limited. This disconnect is driven largely by digital fragmentation across healthcare systems, characterized by poor interoperability between electronic health records, imaging repositories, and genomic databases, as well as by the nontransparent nature of many advanced ML models [11]. The lack of interpretability inherent to “black box” algorithms pose a significant barrier to clinical trust, particularly in oncology, where treatment decisions carry profound consequences for patient outcomes. Consequently, there is an urgent need for integrated, clinically oriented platforms that combine multi-modal data while incorporating explainable artificial intelligence (xAI) methodologies to enhance transparency and clinician confidence [12,13].

Within this context, the present study introduces the methodological framework and objectives of the LANTERN project [14], a multicenter initiative designed to bridge the gap between computational innovation and clinical utility through the development of digital human avatars (DHA). These avatars are imagined as individualized digital representations of patients, constructed through the integration of multi-modal, imaging, and clinical data to model patient-specific disease biology. By leveraging interpretable ML approaches, including gradient-boosted decision trees and SHAP-based explainability, the LANTERN framework aims to generate predictive models for diagnosis, prognosis and treatment response that are both robust and clinically actionable. This project aims to deliver an integrated multi-modal platform for the management of lung cancer patients, enhancing the efficiency in the use of both financial and human resources within healthcare services. The development of DHA is expected to enable a more precise and effective approach to treatment and prevention for these patients. Consequently, substantial benefits are anticipated, including a decrease in healthcare expenditures, improved access to medical services, and a reduction in social disparities. These outcomes will be achieved through shorter patient visit durations, lower costs associated with unnecessary treatments, reduced expenses related to toxicity management, faster patient recovery, and shorter overall hospitalization periods. Moreover, the platform will ensure timely and appropriate healthcare access for all patients, regardless of age, gender, or socioeconomic background. Finally, the advancement of DHA and the integration of predictive models into clinical practice will enhance diagnostic accuracy and support fully personalized treatment strategies.

## 2. Results

Cohort Characteristics and Data Distribution. The study population consisted of 437 patients as the primary cohort diagnosed with clinical N0 (cN0) non-small cell lung cancer (NSCLC) who underwent surgical resection originally described by Ouyang et al. [15]. The prevalence of OLM in the training cohort was 19% (*n* = 82 patients). To assess generalizability, the model was further evaluated on a prospective dataset and an independent external dataset (*n* = 100 patients each), which exhibited slightly higher metastatic rates (23% and 22% respectively). Detailed baseline characteristics, including age, sex, histology, and smoking status, are summarized in the original publication. No significant statistical differences in key clinical variables were observed between the training and validation sets, ensuring the reliability of the external performance assessment.

**Figure 1:**
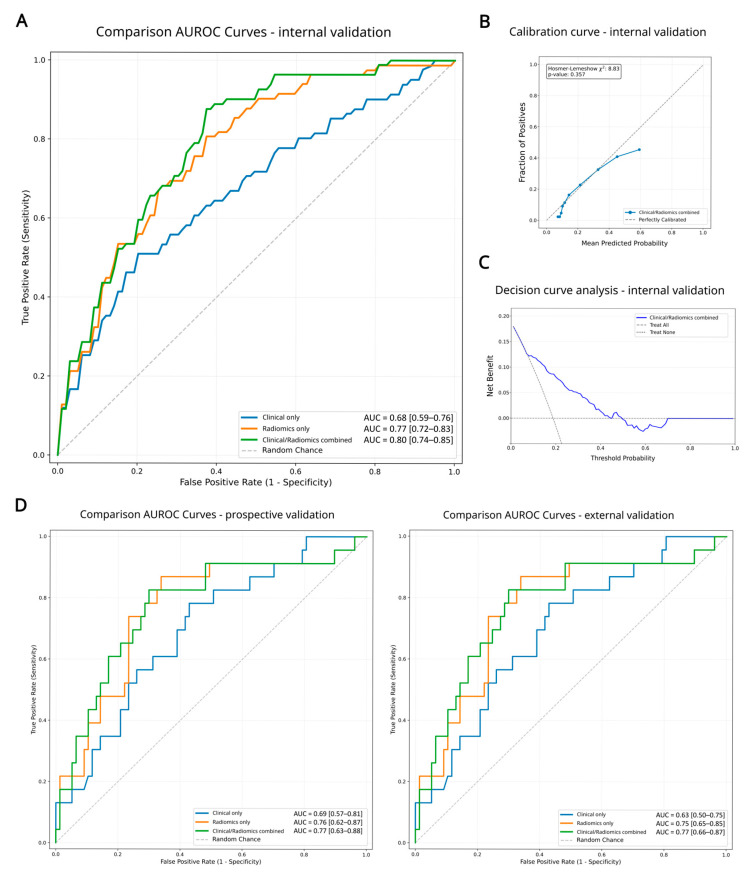
Model Performance and Generalizability Across Internal and External Cohorts. (**A**) Receiver Operating Characteristic (ROC) curves comparing the predictive performance of different data modalities within the internal validation cohort. The plot contrasts the discrimination capabilities of the clinical-only model (blue, AUC = 0.68), the radiomics-only model (orange, AUC = 0.77), and the integrated clinical/radiomics combined model (green). The Combined model demonstrated the highest performance with an Area Under the Curve (AUC) of 0.80. (**B**) Calibration curve for the combined LANTERN-XGB model in the internal validation cohort, assessing the agreement between the model-predicted probabilities of occult lymph node metastasis (*x*-axis) and the actual observed clinical frequencies (*y*-axis). The diagonal dashed line represents perfect algorithmic calibration. (**C**) Decision Curve Analysis (DCA) evaluating the net clinical benefit (*y*-axis) of the LANTERN-XGB framework across a continuum of theoretical risk thresholds (*x*-axis) within the internal validation cohort. The model’s performance is compared against the default clinical strategies of assuming all patients have occult metastasis (“treat all”) or assuming no patients have metastasis (“treat none”). (**D**) ROC curves demonstrating the model’s robustness and generalizability on two independent external datasets. The combined model maintained high predictive accuracy with AUCs of 0.77 (**left**) and 0.77 (**right**), statistically matching the performance benchmarks established in the original study.

Predictive Performance and Benchmarking. The integrated LANTERN-XGB model demonstrated robust discrimination capabilities. In the internal validation cohort, the model achieved an Area Under the Curve (AUC) of 0.80 (Figure 1A and Table 1), performing comparably to the AUC reported for the original radiomics signature. Notably, when compared to the results reported in the original study source, LANTERN-XGB pipeline demonstrated a relative performance increase of 1%, validating the efficacy of the hierarchical feature selection strategy. Critically, in the external validation cohorts this pipeline maintained high performance with an AUC of 0.77 for both external datasets (Figure 1D and Table 1). This result is statistically equivalent to the performance reported for the original external test cohorts. Furthermore, unlike the original nomogram which showed a performance drop when integrating clinical data, this workflow effectively synthesized age, carcinoembryonic antigen (CEA) and squamous cell carcinoma antigen (SCC_Ag) with radiomics to stabilize predictions. Beyond basic discrimination, the clinical applicability of the LANTERN-XGB framework was validated through calibration and decision-making metrics. The calibration curves demonstrated strong concordance between the model’s predicted probabilities and the actual observed frequencies of OLM, indicating that the predicted risk scores are reliable representations of true metastatic risk (Figure 1B and Supplementary Figure S1A). Furthermore, Decision Curve Analysis (DCA) revealed that the LANTERN-XGB model provided a superior net clinical benefit compared to both “treat all” and “treat none” baseline strategies across a wide range of clinically relevant threshold probabilities (Figure 1C and Supplementary Figure S1B). Utilizing the optimal decision threshold derived from the training cohort, the model achieved a sensitivity of 73.9% and 68.2% and a specificity of 72.7% and 75.6% in the prospective and external validation datasets respectively. A comprehensive comparison of these and other predictive metrics between the LANTERN-XGB framework and the original work is detailed in Supplementary Table S1. This balanced performance demonstrates the framework’s capacity to accurately identify high-risk patients requiring invasive staging while safely minimizing false-positive escalations.

Exploratory Pathway Correlates of Model Predictions. To decode the biological mechanisms driving the model’s risk predictions, we evaluated the correlation between the LOO-predicted probabilities and the GSVA hallmark pathway enrichment scores within the transcriptomic subset (Supplementary Table S2). Rather than relying on isolated gene expressions, which are susceptible to small-sample biases, this pathway-level approach revealed a robust systemic signature. Specifically, higher model-predicted risk scores (indicating a greater probability of OLM) were significantly correlated with the positive enrichment of key immune and inflammatory pathways, including Interferon-Gamma Response (r = 0.66, adjusted *p*-value = 0.025), Interferon-Alpha Response, and IL2-STAT5 Signaling. This independently corroborates previous findings linking OLM to immune-centric modules. Furthermore, high-risk predictions were strongly associated with metabolic stress and hyperproliferation pathways, notably the Unfolded Protein Response (r = 0.69, adjusted *p*-value = 0.018), G2M Checkpoint, and MYC/mTORC1 signaling. Conversely, lower predicted risk probabilities were characterized by a strong negative correlation with tissue differentiation signatures, such as Myogenesis (r = −0.77, adjusted *p*-value = 0.003).

**Figure 2:**
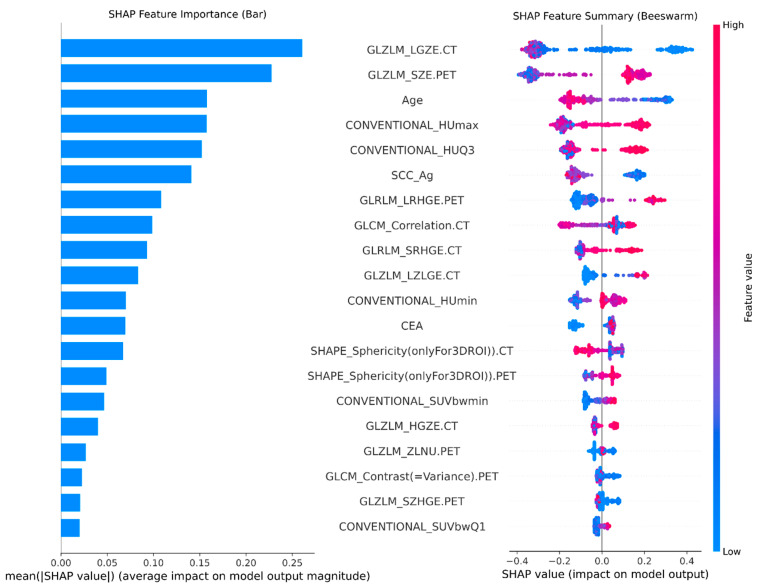
Global Interpretability and Multi-Modal Consensus Signature. (**Left**) A SHAP feature importance bar plot ranking the top features by their mean absolute impact on model output magnitude. The hierarchical selection process identified a robust consensus signature comprising nine key features, demonstrating synergistic interactions between CT radiomics (e.g., GLZLM_LGZE.CT), PET metabolic features (e.g., GLZLM_SZE.PET), and clinical Age. These variables were retained as stable predictors across independent cross-validation folds to mitigate overfitting. (**Right**) A SHAP beeswarm summary plot illustrating the directionality of feature effects across the cohort. Each dot represents an individual patient, where red indicates a high feature value and blue indicates a low feature value. For instance, high values of GLZLM_SZE.PET (red) consistently correlate with positive SHAP values, indicating a direct association with increased metastatic risk. Conversely, the plot reveals how lower values of specific textural markers or clinical modulators can serve as protective signals within the individual patient profile.

Identification of Key Multi-Modal Features. The hierarchical XGBoost-based feature selection process identified a robust “consensus signature” comprising 20 key features, spanning clinical, morphological, and textural domains. As detailed in Figure 2, this selection partially overlaps with the radiomic signature identified in the original study, providing cross-methodological validation of the underlying biological signal, while also capturing distinct novel predictors. Regarding CT Radiomics, consistent with the original findings, the LANTERN-XGB model identified GLZLM_LZLGE.CT as a stable marker. However, our pipeline revealed that GLZLM_LGZE.CT (Large Zone Low Gray-level Emphasis) was the most dominant overall predictor of occult metastasis. Furthermore, while the benchmark study relied solely on CONVENTIONAL_HUmax (maximum density), our model selected both CONVENTIONAL_HUmax and CONVENTIONAL_HUQ3. This suggests that the broader distribution of high-density tissue, combined with 3D morphological constraints like SHAPE_Sphericity, provides a more comprehensive measure of tumor invasiveness. Metabolic heterogeneity was powerfully captured through GLZLM_SZE.PET (Small Zone Emphasis), reflecting a fragmented metabolic architecture biologically associated with rapid proliferation and hypoxia.

Finally, in stark contrast to the original study which found clinical features to be non-contributory, the LANTERN-XGB model successfully integrated systemic data. Age emerged as a top-tier predictor, acting alongside specific blood biomarkers, notably SCC_Ag and CEA. This demonstrates that while clinical and serological variables may lack strong linear relationships with metastasis in isolation, they act as critical non-linear modulators of risk within a multi-modal machine learning framework.

**Figure 3:**
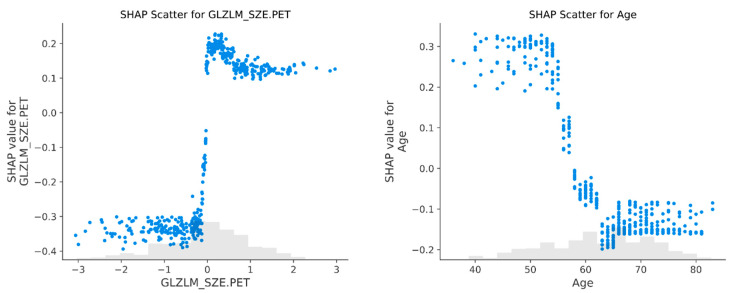
Clinical Threshold Discovery via SHAP Dependence Plots. Scatter plots visualizing the non-linear relationships between feature values (*x*-axis) and their contribution to metastatic risk (SHAP value, *y*-axis). The gray shaded area in the background of each plot represents the histogram of the sample distribution, indicating the frequency of patients at each corresponding feature value. (**Left**) The dependence plot for GLZLM_SZE.PET identifies a risk threshold at value −0.04, where risk scores increase sharply. (**Right**) The dependence plot for Age reveals a “safe zone” for patients older than 57.5 years, demonstrating how the model uses age as a non-linear modulator of risk rather than a linear predictor.

Global Model Interpretability and Biomarker Ranking. To understand the biological logic driving the model’s predictions, the global SHAP values were analyzed. Figure 3 displays the SHAP summary beeswarm plot, ranking features by their mean absolute impact on the model output. The analysis revealed that GLZLM_LGZE.CT, GLZLM_SZE.PET and Age were the top contributors to metastatic risk. High values of GLZLM_SZE.PET and low level of GLZLM_LGZE.CT and younger patients were consistently associated with positive SHAP values, indicating an increased likelihood of occult metastasis. Interestingly, while radiomic features dominated the top rankings, clinical variables such as Age and SCC_Ag provided critical adjustments to the risk scores for patients with ambiguous imaging phenotypes, highlighting the necessity of the multi-modal approach.

Translation to Clinical Thresholds To facilitate clinical utility, decision-tree stumps were utilized to convert the continuous relationships learned by the model into actionable cutoffs (Figure 3). For example, the dependence plot for GLZLM_SZE.PET revealed a non-linear threshold at value −0.04; patients exceeding this threshold exhibited a sharp increase in metastatic risk score. Similarly, the model identified a “safe zone” for patients with Age above 57.5, effectively stratifying the population into clear low-risk and high-risk groups that align with clinical intuition.

**Figure 4:**
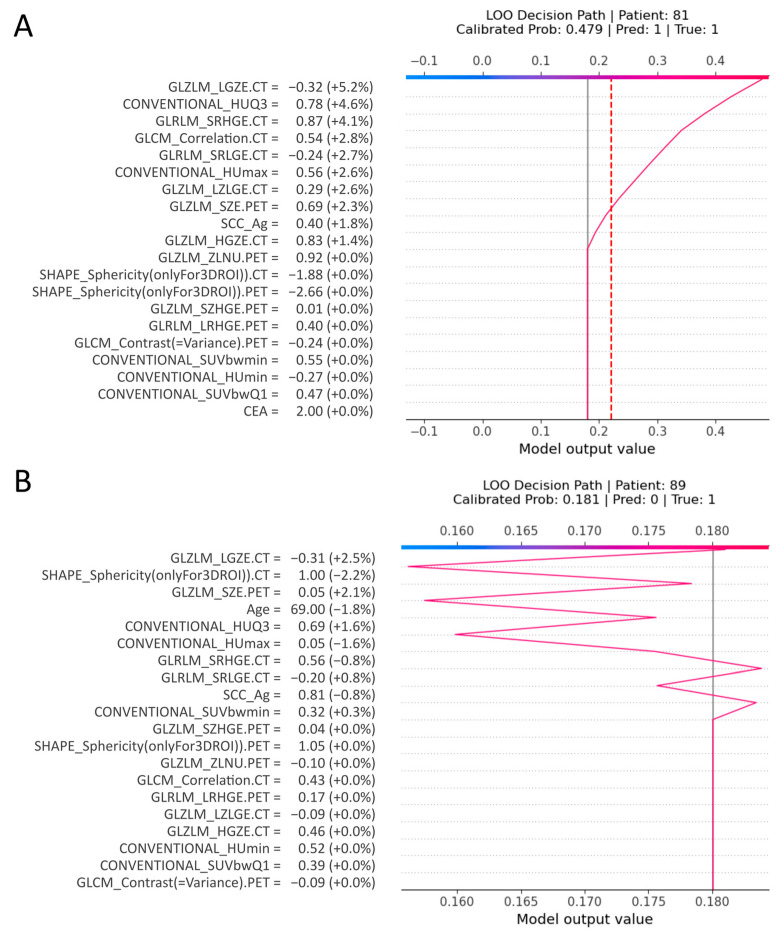
Local Interpretability: The Digital Human Avatar in Practice. SHAP force plots visualizing the decision paths for two specific patients from the validation cohort. The solid gray line represents the baseline average risk (base value) of the entire cohort, while the red dotted line represents the diagnostic threshold (22.1%). The colored trajectories track the individual patient’s cumulative prediction path, demonstrating how each specific feature value pushes the risk score away from the baseline. (**A**) A “True Positive” case where aggressive radiomic features coherently push the model output toward a high probability of metastasis. (**B**) A “False Negative” scenario where, despite some protective signals, aggressive risk factors are visualized. This allows the clinician to interpret the biological logic and potentially reclassify the patient as high risk, preventing potential metastasis and subsequent undertreatment.

**Local Interpretability: The Digital Human Avatar in Practice.** To demonstrate the clinical utility of the LANTERN-XGB workflow, the decision paths for two representative patients from the external validation cohort were examined using SHAP force plots (Figure 4). These visualizations are particularly critical for “borderline” cases where the model’s probability score hovers near the decision threshold (approximately 18%), rendering a binary “High/Low Risk” output insufficient for clinical confidence. In these scenarios, the Digital Human Avatar allows the clinician to deconstruct the prediction, effectively opening the “black box” to validate whether the algorithmic logic aligns with medical experience. The patient in Figure 4A represents a clear “true positive” scenario. The model confidently predicted the “presence” of occult metastasis. The force plot confirms that this high-risk classification was not an artifact but was driven by a coherent set of aggressive radiomic features that strongly pushed the probability score upward. In contrast, the patient in Figure 4B illustrates the critical “human-in-the-loop” advantage. In this case, the model initially predicted a negative result (absence of OLM, probability score 0.181), which would typically lead to a waiver of invasive staging. However, this prediction was a False Negative. By exploring the local force plot, the clinician could deconstruct this erroneous signal. While certain protective “counter-forces”, specifically the patient’s older Age (69 years) and high tumor sphericity (SHAPE_Sphericity.CT = 1.00), drove the risk score down, the visualization revealed significant underlying risk factors. The plot explicitly highlighted aggressive markers pushing the risk upward, notably CT textural fragmentation (GLZLM_LGZE.CT), PET metabolic heterogeneity (GLZLM_SZE.PET), and high third-quartile density (CONVENTIONAL_HUQ3). Recognizing that these specific markers of biological aggressiveness were more clinically alarming in this context than the protective structural features, the clinician could choose to trust these aggressive signals more heavily. This interpretability allows for a reasoned disagreement with the raw probability score, upgrading the patient to “High Risk” (presence of OLM) and effectively preventing a missed metastasis and subsequent undertreatment.

## 3. Discussion

In this study, we successfully implemented and validated the LANTERN-XGB workflow, a comprehensive multi-modal framework designed to bridge the translational gap in non-small cell lung cancer (NSCLC) staging. Our findings demonstrate that the LANTERN-XGB pipeline is not only technically robust but also clinically aligned with current state-of-the-art benchmarks. By benchmarking our approach against established literature and external datasets, we confirmed that the model delivers predictive performance that is consistent with, and in several metrics’ superior to, existing methodologies. Personalized medicine represents an evolution from traditional “one size fits all” strategies, extending the concept of stratifying cancer treatments on well-known clinical and pathological characteristics to a more individual, patients specific, truly personalized level. However, the current approach typically relies on molecular and cellular datasets (i.e., specific mutations) that are often limited to narrow subsets of the complex cellular networks determining treatment outcomes. Due to various histological, genetic, immunological, and imaging characteristics that define its highly variable oncological outcomes, lung cancer represents an ideal case to run AI based multidimensional personalized medicine studies, thereby fitting into the purpose of LANTERN project. Indeed, this initiative has the ambition to combine data from macroscopic imaging and biological features by combining radiomic features with high-throughput multi-modal data—ranging from genomic landscapes (including somatic mutations, copy number variations, signature as TMB and MSI) to transcriptomic profiles—on a unified digital platform. Additionally, by integrating NGS-derived data, such as driver mutations and co-mutational profile, the LANTERN-XGB workflow can better account for intratumoral heterogeneity and the evolutionary trajectory of the disease. The application of this platform will allow the development of innovative technological prototypes, based on the inclusion of various human biological functions to obtain quantitative models capable of improving the understanding the physio-pathological processes, mechanisms under lung oncogenesis, including cellular signaling dysregulation and immune checkpoint expression, in patient with lung tumor. Ultimately, the development of Digital Human Avatars, informed by the patient’s specific genotype-phenotype correlations, and the consequent application of predictive models in clinical practice will in fact enhance the accuracy of diagnosis and a complete personalization of treatment.

The distinguishing characteristic of this work, however, lies beyond raw performance metrics. By formalizing the concept of the “Digital Human Avatar”, we have moved from opaque “black-box” probability scores to a transparent, interpretable decision-support system. The workflow provides clinicians with granular insights through intuitive plots and tables, specifically SHAP force plots, which decompose the risk for each individual patient. This interpretability allows medical professionals to explore “borderline” cases, verify that the model’s logic aligns with biological intuition (e.g., the protective role of age, presence of genomic mutations or specific texture patterns), and ultimately make more informed decisions regarding invasive staging. To adopt collaboration and accelerate the implementation of these tools in the broader scientific community, the complete pipeline, including the feature selection and visualization modules, has been made freely available as an open-source resource.

While the current implementation of the LANTERN-XGB clinical reporting module relies on a deterministic, hard-coded layer to maximize clinical safety and reproducibility, this approach inherently limits the breadth of the narrative. A highly promising future direction involves the integration of advanced Large Language Models (LLMs) to facilitate dynamic clinical report generation. Responsibly fine-tuned LLMs could synthesize complex, multi-modal SHAP interactions into highly nuanced patient narratives, continuously cross-referencing the patient’s “Digital Human Avatar” against rapidly evolving oncological literature. Such integration would transition the pipeline from static interpretations to an adaptive, omni-comprehensive diagnostic assistant, provided that strict guardrails against AI hallucinations are maintained in the clinical setting.

Despite the robust predictive performance achieved through clinical and radiomic integration, we acknowledge that the transcriptomic component of our study is constrained by the small sample size of the radiogenomic cohort (*n* = 20). Furthermore, as an exploratory bulk RNA-sequencing analysis, these findings are inherently susceptible to biases arising from intratumoral heterogeneity, variations in library preparation, and sequencing platform differences. Consequently, the GSVA pathway signatures we report serve strictly as hypothesis-generating biological correlates rather than definitive, standalone diagnostic markers. However, the significant pathway-level correlations observed between our model’s risk predictions and underlying molecular dysregulation highlight a critical imperative for the future of precision oncology. These results strongly advocate for the integration of routine transcriptomic profiling into standard clinical diagnostic workflows. By making the acquisition of these molecular layers a routine clinical practice, future large-scale prospective trials will be fully equipped to validate multi-modal digital avatars, ultimately bridging the gap between macroscopic imaging phenotypes and actionable molecular targets.

It is important to distinguish between the benchmarking phase of this study and the ongoing prospective goals of the LANTERN initiative. The use of the retrospective cohort originally described by Ouyang et al. was a deliberate methodological choice to ensure a rigorous and direct comparison between the LANTERN-XGB framework and established state-of-the-art results. By utilizing this standardized historical data, we were able to quantify a relative performance increase and validate the stability of our hierarchical feature selection. Despite these promising advances, several limitations must be acknowledged. While we performed external validation, the multi-centric nature of radiomics data collection introduces variability due to differences in scanner protocols, reconstruction kernels, and contrast timing. Although our pipeline includes preprocessing steps to mitigate these “batch effects,” complete standardization remains a challenge for the field. Moreover, while SHAP values provide mathematical transparency, the biological translation of complex high-order texture features (e.g., GLZLM_SZE) remains abstract. Future translational research is required to correlate these radiomic phenotypes with specific histopathological microenvironments or transcriptomic signatures to fully validate their biological significance.

## 4. Materials and Methods

Study Cohort and Dataset To evaluate the efficacy of the proposed pipeline, a retrospective dataset was employed comprising clinical and radiomics data derived from a previously published cohort of 437 patients [15]. This dataset integrates 20 clinical features with 128 radiomic features from PET/CT. To assess the model’s generalizability and robustness, validation was performed using two independent external datasets, each consisting of 100 patients, maintaining the same feature set availability. Critically, for a specific radiogenomic cohort (RGC) consisting of 20 patients, matched high-throughput RNA sequencing data providing expression profiles for both lncRNA and mRNA were available. This subset enabled the integration of transcriptomic layers into the LANTERN-XGB workflow to identify synergistic molecular drivers. The primary classification task for this study was the prediction of occult lymph node metastasis (OLM), with results benchmarked against those reported in the original publication to quantify potential diagnostic improvements.

Data Integration and Preprocessing The analytical framework was developed using the Python v3.13 programming language and is publicly accessible via the LANTERN GitHub repository (The pipeline is implemented to process and integrate diverse, heterogeneous multi-modal tabular data, ranging from clinical and spirometric records to genomic signature and radiomic profiles. Data processing is managed through a modular loading system that executes outer joins across different modalities based on unique patient identifiers, ensuring maximal retention of available information while preserving data integrity. Pipeline execution is controlled through a user-configurable settings file that enables reproducible customization of preprocessing steps, feature subsets, and modeling parameters. This modular architecture supports systematic experimentation across unimodal and multimodal configurations, enabling comparative evaluation of predictive performance across data modalities and facilitating empirical determination of the most informative integrative strategy.

Cross-Center Data Harmonization. Radiomic features are inherently sensitive to variations in scanner protocols, reconstruction algorithms, and center-specific acquisition parameters. To mitigate batch effects and ensure the real-world applicability of the LANTERN-XGB pipeline across multi-centric datasets, a robust data harmonization module was integrated into the preprocessing workflow. When evaluating prospective or external cohorts, the pipeline applies ComBat [16,17] to harmonize feature distributions and align the external data with the feature space of the training cohort. To rigorously validate this process and simulate real-world clinical inference, a Leave-One-Out (LOO) harmonization simulation was implemented. In this simulation, the dataset is harmonized using the statistical parameters of the specific batch while explicitly excluding the single test patient. The derived transformation is then applied to the test patient, thereby preventing data leakage and ensuring that the model’s performance metrics accurately reflect its capability to handle data from diverse clinical environments.

### The Multi-Stage XGBoost Workflow

**Figure 5:**
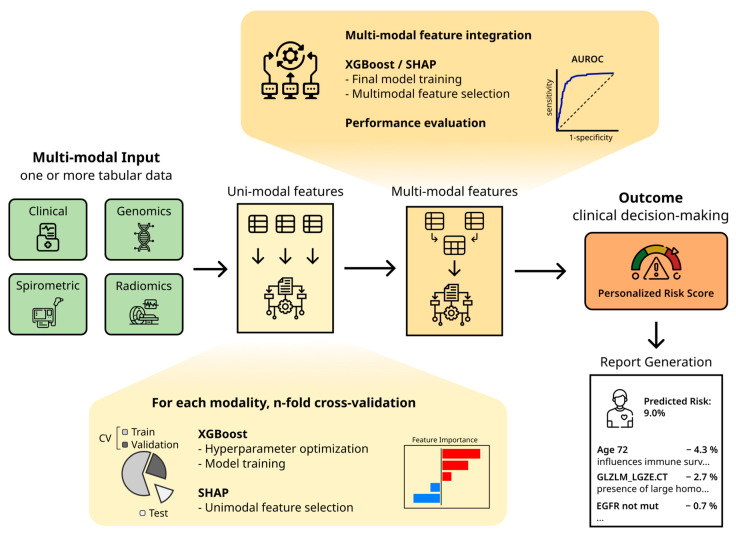
The LANTERN-XGB Workflow Architecture. This schematic illustrates the hierarchical machine learning pipeline designed to generate “Digital Human Avatars” for lung cancer staging. The workflow inputs heterogeneous multi-modal input data (clinical, genomics, spirometric, and/or radiomics) and processes it through a multi-stage architecture. Unimodal features undergo independent selection using XGBoost and SHAP within a cross-validation scheme before aggregation into a multimodal dataset. The final output is a personalized risk score for binary/multiclass or survival outcome, incorporating interpretable visualizations and a report to support clinical decision-making.

The analytical core of this study is a hierarchical, multi-stage workflow (Figure 5) based on XGBoost (v3.2) [18]. This framework was selected for its high computational efficiency, resilience to feature collinearity, and intrinsic capability to manage missing values, a pervasive challenge in routine clinical data, thereby facilitating the robust analysis of incomplete patient profiles without reliance on external imputation. To ensure a rigorous evaluation of model generalizability, the workflow implements a n-fold stratified cross-validation scheme. To mitigate data leakage, feature selection and model training were confined strictly to training partitions within each fold, producing consensus feature sets that were subsequently evaluated on held-out test folds. Hyperparameter optimization was performed using Bayesian search (BayesSearchCV), targeting optimization of learning rate, maximum tree depth, and subsampling ratios, consistent with best practices in ML optimization.

Two-Tier Feature Selection Strategy. To address the dimensionality imbalance between high-throughput modalities (e.g., radiomics or transcriptomics) and standard clinical datasets, a two-tiered feature selection strategy was implemented. This approach is designed to mitigate the risk of overfitting and prevent high-dimensional data from overshadowing smaller, yet clinically significant, feature sets.

In the first stage, unimodal feature selection was performed independently for each data modality using nested n-fold cross-validation. Feature importance rankings were computed using SHapley Additive exPlanations (SHAP), a game-theoretic interpretability framework widely adopted for model explanation in biomedical machine learning [19]. To enhance the robustness of biomarker discovery, features were evaluated using a dual-metric approach that combines their mean SHAP importance with a dedicated stability score. This stability score quantifies the frequency and consistency of a feature’s high-ranking contribution across all cross-validation folds. Features were retained for the subsequent multimodal aggregation phase only if they exceeded optimized thresholds for both importance and stability, thereby guaranteeing that the final set of modality-specific predictors is not only highly influential but also strictly generalizable across data permutations.

In the second stage, all retained unimodal features were aggregated into a unified multimodal dataset. A secondary selection step was then applied to identify synergistic cross-modal predictors, ensuring that the final model captured robust interactions among heterogeneous biological and clinical signals, in alignment with emerging best practices in integrative oncology modeling [20].

Management of Missingness. Recognizing that missing data in clinical practice may represent a systematic non-random event rather than a random occurrence [21], the pipeline includes a specific module for the exploration of missingness. If the frequency of missing values for a specific feature exceeds a user-defined threshold, the pipeline generates a new binary feature (e.g., smoking_ismissing) while imputing the original variable. This allows the model to assess whether the absence of data itself acts as a predictor. For instance, if smoking status is systematically unrecorded for patients with early-stage disease, perhaps due to less aggressive clinical profiling, the smoking_ismissing flag might erroneously become a strong predictor of favorable outcomes. If missingness indicators emerged as dominant drivers of prediction during feature selection, both the indicator and the corresponding original feature were removed to prevent bias arising from non-random data acquisition patterns.

Model Interpretability (xAI). To ensure transparency and facilitate clinical translation, model interpretability was implemented using SHAP-based explainable artificial intelligence techniques. A dual-layer interpretability strategy was implemented addressing both population-level validation and patient-specific decision support.

Clinical Utility and Global Model Evaluation. To rigorously evaluate the clinical utility and global calibration of the LANTERN-XGB model, performance was assessed beyond standard AUC metrics. Model calibration was evaluated using calibration curves (reliability diagrams) to compare the predicted probabilities against the observed clinical frequencies. To assess the practical value of the model in a real-world setting, Decision Curve Analysis (DCA) was performed. DCA quantifies the net clinical benefit of the predictive model across a continuum of theoretical risk thresholds, comparing it against default strategies (e.g., treating all patients or treating no patients). Furthermore, to translate continuous risk probabilities into actionable binary classifications, an optimal clinical decision threshold was determined using Youden’s J statistic on the training cohort. Model accuracy, sensitivity and specificity were subsequently calculated at this optimized cutoff for all validation cohorts.

Global Interpretability and A Priori Validation. At the global level, interpretability allows for an a priori assessment of the model’s biological plausibility before clinical deployment [22]. Summary beeswarm plots were generated to elucidate the overall impact and directionality of features across the entire cohort, ensuring that the model’s learned associations align with established medical literature. Furthermore, to translate complex algorithmic logic into actionable clinical heuristics, decision-tree-based “stumps” was applied to analyze the relationship between feature values and their SHAP contributions. This process identifies non-linear inflection points, effectively converting continuous variables into clinically relevant stratification thresholds (e.g., defining an optimal cutoff such as “Age < 55.5”, specific radiomic intensity levels or specific expression levels) [23,24].

Local Explanations and Clinical Decision Support. At the granular, single-patient level, local interpretability is utilized to decompose the specific drivers of an individual prediction. Through the use of “force plots”, the pipeline visualizes how specific biomarkers synergize to increase or decrease a patient’s specific risk score. Furthermore, Individual Conditional Expectation (ICE) plots are integrated to enable dynamic “what-if” scenario analyses. ICE plots illustrate how an individual patient’s predicted risk would shift if a specific feature value were altered, while holding all other variables constant. This functionality is particularly critical for “borderline” patients whose probability scores hover near the decision threshold. By simulating variations in a key feature, such as adjusting a radiomic texture value that might be susceptible to scanner noise, clinicians can immediately assess the fragility or robustness of the prediction. This empowers the treating physician to verify that the model’s output is supported by concrete, stable clinical values, serving as an intelligent second opinion rather than an opaque directive [25]. Finally, these visual explanations facilitate shared decision-making; by clearing the “why” behind a prediction, they provide an intuitive channel for clinicians to explain complex data-driven treatment strategies to patients, thereby fostering greater engagement and transparency in the therapeutic process [26].

Automated Clinical Report Generation. While force plots provide mathematical transparency regarding feature contributions, translating these algorithmic outputs into actionable clinical insights can remain challenging in routine practice. To bridge this gap, the LANTERN-XGB pipeline incorporates an automated Clinical Report Generator. This module automatically translates raw SHAP contribution scores and patient-specific predictions into a readable, individualized PDF narrative. To guarantee clinical safety and ensure high reproducibility, the reporting module utilizes a deterministic interpretation layer. This layer employs hard-coded, literature-vetted biological and anatomical explanations to contextualize the most prominent features (e.g., mapping specific PET metabolic heterogeneity metrics to underlying tumor aggressiveness). This ensures that the generated digital human avatars provide clinicians with a reliable, transparent, and biologically grounded decision-support narrative.

Transcriptomic Pathway Analysis (GSVA). To mitigate the noise, overfitting risks, and biases inherent to single-gene analysis in small bulk RNA-sequencing cohorts, the transcriptomic evaluation was shifted to a robust pathway-level approach. Gene Set Variation Analysis (GSVA) [27] was conducted to compute sample-wise enrichment scores for all established hallmark gene sets from the Molecular Signatures Database (MSigDB) [28]. To investigate the biological correlates underlying the algorithm’s multi-modal predictions, these patient-specific GSVA scores were statistically correlated with the output predicted probabilities generated from the LOO analysis of the prospective and external datasets. This methodology effectively aggregates individual transcriptomic variances into stable, biologically interpretable pathway signatures linked directly to the model’s risk assessments.

## 5. Conclusions

In summary, the LANTERN-XGB framework represents a validated, open-access, and highly interpretable methodology for precision oncology. By empowering clinicians to visualize and query the logic behind AI predictions, it offers a pragmatic path toward the integration of computational intelligence into the daily routine of lung cancer care, paving the way for future prospective clinical trials. When applied to liquid biopsy, the current framework may fully capture also the dynamic clonal evolution of the tumor or the transient changes in the circulating tumor DNA (ctDNA) fraction under therapeutic pressure.

## Figures and Tables

**Table 1:** Predictive Performance Benchmarking. A comparison of Area Under the Curve (AUC) metrics between the original benchmark study (OG) and the LANTERN-XGB workflow (L-XGB) following cross-center data harmonization and Leave-One-Out (LOO) validation. The table presents performance data with 95% Confidence Intervals (CI) for clinical-only, radiomics-only, and combined multi-modal models across the internal validation cohort, a prospective test cohort, and an independent external test cohort. The XGBoost-based pipeline demonstrates robust and comparable performance to the benchmark, effectively stabilizing predictions on unseen data by integrating clinical features with harmonized radiomics (Combined AUC = 0.80 internal, AUC = 0.77 prospective, and AUC = 0.77 external).

		Clinical	Radiomics	Combined
Internal validation				
OG	AUC (95% CI)	0.59 (0.46–0.72)	0.81 (0.71–0.91)	0.79 (0.69–0.90)
L-XGB	AUC (95% CI)	0.68 (0.59–0.76)	0.77 (0.72–0.83)	0.80 (0.74–0.85)
Prospective test				
OG	AUC (95% CI)	0.60 (0.46–0.73)	0.80 (0.69–0.90)	0.76 (0.65–0.88)
L-XGB	AUC (95% CI)	0.69 (0.57–0.81)	0.76 (0.62–0.87)	0.77 (0.63–0.88)
External test				
OG	AUC (95% CI)	0.68 (0.55–0.81)	0.78 (0.67–0.88)	0.79 (0.68–0.90)
L-XGB	AUC (95% CI)	0.63 (0.50–0.75)	0.75 (0.65–0.85)	0.77 (0.66–0.87)

## Data Availability

Data available upon authors request.

## Supplementary Materials

The following supporting information can be downloaded at: https://www.mdpi.com/article/10.3390/ijms27073128/s1.

## References

[1] Bray F., Laversanne M., Sung H., Ferlay J., Siegel R.L., Soerjomataram I., Jemal A. (2024). Global Cancer Statistics 2022: GLOBOCAN Estimates of Incidence and Mortality Worldwide for 36 Cancers in 185 Countries. CA. Cancer J. Clin..

[2] Siegel R.L., Giaquinto A.N., Jemal A. (2024). Cancer Statistics, 2024. CA. Cancer J. Clin..

[3] Li J., Wu J., Zhao Z., Zhang Q., Shao J., Wang C., Qiu Z., Li W. (2021). Artificial Intelligence-Assisted Decision Making for Prognosis and Drug Efficacy Prediction in Lung Cancer Patients: A Narrative Review. J. Thorac. Dis..

[4] Zhong Y., She Y., Deng J., Chen S., Wang T., Yang M., Ma M., Song Y., Qi H., Wang Y. (2022). Deep Learning for Prediction of N_2_ Metastasis and Survival for Clinical Stage I Non-Small Cell Lung Cancer. Radiology.

[5] Jin L., Zhao Q., Fu S., Cao F., Hou B., Ma J. (2023). Development and Validation of Machine Learning Models to Predict Survival of Patients with Resected Stage-III NSCLC. Front. Oncol..

[6] She Y., Jin Z., Wu J., Deng J., Zhang L., Su H., Jiang G., Liu H., Xie D., Cao N. (2020). Development and Validation of a Deep Learning Model for Non-Small Cell Lung Cancer Survival. JAMA Netw. Open.

[7] Coudray N., Ocampo P.S., Sakellaropoulos T., Narula N., Snuderl M., Fenyö D., Moreira A.L., Razavian N., Tsirigos A. (2018). Classification and Mutation Prediction from Non-Small Cell Lung Cancer Histopathology Images Using Deep Learning. Nat. Med..

[8] Zhang Y., Wang S., Chen X., Zhang G., Wang Y., Liu X. (2025). A 23-Gene Multi-Omics Signature Predicts Prognosis and Treatment Response in Non-Small Cell Lung Cancer. Discov. Oncol..

[9] Kinoshita F., Takenaka T., Yamashita T., Matsumoto K., Oku Y., Ono Y., Wakasu S., Haratake N., Tagawa T., Nakashima N. (2023). Development of Artificial Intelligence Prognostic Model for Surgically Resected Non-Small Cell Lung Cancer. Sci. Rep..

[10] Christopoulos P., Harel M., McGregor K., Brody Y., Puzanov I., Bar J., Elon Y., Sela I., Yellin B., Lahav C. (2024). Plasma Proteome-Based Test for First-Line Treatment Selection in Metastatic Non-Small Cell Lung Cancer. JCO Precis. Oncol..

[11] Miotto R., Wang F., Wang S., Jiang X., Dudley J.T. (2018). Deep Learning for Healthcare: Review, Opportunities and Challenges. Brief. Bioinform..

[12] Abas Mohamed Y., Ee Khoo B., Shahrimie Mohd Asaari M., Ezane Aziz M., Rahiman Ghazali F. (2025). Decoding the Black Box: Explainable AI (XAI) for Cancer Diagnosis, Prognosis, and Treatment Planning-A State-of-the Art Systematic Review. Int. J. Med. Inf..

[13] Ladbury C., Zarinshenas R., Semwal H., Tam A., Vaidehi N., Rodin A.S., Liu A., Glaser S., Salgia R., Amini A. (2022). Utilization of Model-Agnostic Explainable Artificial Intelligence Frameworks in Oncology: A Narrative Review. Transl. Cancer Res..

[14] Lococo F., Boldrini L., Diepriye C.-D., Evangelista J., Nero C., Flamini S., Minucci A., De Paolis E., Vita E., Cesario A. (2023). Lung Cancer Multi-Omics Digital Human Avatars for Integrating Precision Medicine into Clinical Practice: The LANTERN Study. BMC Cancer.

[15] Ouyang M.-L., Yao Y.-Z., Xia H.-W., Tang K., Zuo Z.-Y., Wang L.-L., Lin J., Li J., Huang X.-Y., Wang L.-X. (2025). Imaging and RNA Sequencing-Based Radiogenomic Analysis of Occult Lymph Node Metastasis and Survival in Lung Adenocarcinoma Staged Non-Metastatic. Commun. Med..

[16] Johnson W.E., Li C., Rabinovic A. (2007). Adjusting Batch Effects in Microarray Expression Data Using Empirical Bayes Methods. Biostat. Oxf. Engl..

[17] Fortin J.-P., Cullen N., Sheline Y.I., Taylor W.D., Aselcioglu I., Cook P.A., Adams P., Cooper C., Fava M., McGrath P.J. (2018). Harmonization of Cortical Thickness Measurements across Scanners and Sites. NeuroImage.

[18] Chen T., Guestrin C. (2016). XGBoost: A Scalable Tree Boosting System. Proceedings of the 22nd ACM SIGKDD International Conference on Knowledge Discovery and Data Mining.

[19] Lundberg S., Lee S.-I. (2017). A Unified Approach to Interpreting Model Predictions. Proceedings of the 31st International Conference on Neural Information Processing Systems.

[20] Lipkova J., Chen R.J., Chen B., Lu M.Y., Barbieri M., Shao D., Vaidya A.J., Chen C., Zhuang L., Williamson D.F.K. (2022). Artificial Intelligence for Multimodal Data Integration in Oncology. Cancer Cell.

[21] Sperrin M., Martin G.P., Sisk R., Peek N. (2020). Missing Data Should Be Handled Differently for Prediction than for Description or Causal Explanation. J. Clin. Epidemiol..

[22] Lundberg S.M., Nair B., Vavilala M.S., Horibe M., Eisses M.J., Adams T., Liston D.E., Low D.K.-W., Newman S.-F., Kim J. (2018). Explainable Machine-Learning Predictions for the Prevention of Hypoxaemia during Surgery. Nat. Biomed. Eng..

[23] Keyl J., Keyl P., Montavon G., Hosch R., Brehmer A., Mochmann L., Jurmeister P., Dernbach G., Kim M., Koitka S. (2025). Decoding Pan-Cancer Treatment Outcomes Using Multimodal Real-World Data and Explainable Artificial Intelligence. Nat. Cancer.

[24] Alharbi W., Alfayez A.A. (2025). Explainable Artificial Intelligence in Pancreatic Cancer Prediction: From Transparency to Clinical Decision-Making. Front. Oncol..

[25] Tonekaboni S., Joshi S., McCradden M.D., Goldenberg A. (2019). What Clinicians Want: Contextualizing Explainable Machine Learning for Clinical End Use. Proceedings of the 4th Machine Learning for Healthcare Conference.

[26] Diprose W.K., Buist N., Hua N., Thurier Q., Shand G., Robinson R. (2020). Physician Understanding, Explainability, and Trust in a Hypothetical Machine Learning Risk Calculator. J. Am. Med. Inform. Assoc. JAMIA.

[27] Hänzelmann S., Castelo R., Guinney J. (2013). GSVA: Gene Set Variation Analysis for Microarray and RNA-Seq Data. BMC Bioinform..

[28] Subramanian A., Tamayo P., Mootha V.K., Mukherjee S., Ebert B.L., Gillette M.A., Paulovich A., Pomeroy S.L., Golub T.R., Lander E.S. (2005). Gene Set Enrichment Analysis: A Knowledge-Based Approach for Interpreting Genome-Wide Expression Profiles. Proc. Natl. Acad. Sci. USA.

