# Supplementary material for: LANTERN-XGB: An Interpretable Multi-Modal Machine Learning for Improving Clinical Decision-Making in Lung Cancer
Source: Int J Mol Sci. 2026 Mar 30;27(7):3128. doi: 10.3390/ijms27073128 (PMC13074020; doi:10.3390/ijms27073128)
Supplement: Supplementary file 1 [file ijms-27-03128-s001.zip › ijms-4188010-supplementary.pdf]

LANTERN-XGB: An Interpretable Multi-Modal Machine Learning for Improving Clinical Decision-Making in Lung Cancer.

Supplementary Material

Supplementary Table S1. Predictive Performance Benchmarking.

Supplementary Table S2. Gene Set Variation Analysis (GSVA) Pathway Correlations with LANTERN-XGB Risk Predictions.

Supplementary Figure S1 Clinical Utility and Calibration of the LANTERN-XGB Model.

Clinical Risk Assessment Report: Patient 81 – Example High Risk Patient (True Positive)

Clinical Risk Assessment Report: Patient 89 – Example Borderline/Low Risk Patient (False Negative)

Supplementary Table S1. Predictive Performance Benchmarking. A detailed comparison of diagnostic performance metrics between the original benchmark study (OG) and the LANTERN-XGB workflow (L-XGB). The table presents Accuracy, Sensitivity, and Specificity for the clinical-only, radiomics-only, and combined multi-modal models. Results are evaluated across three distinct datasets: the internal validation cohort, a prospective test cohort, and an independent external test cohort. The LANTERN-XGB combined model demonstrates balanced and robust performance, particularly highlighting its capacity to maintain stable sensitivity and specificity during external and prospective validation, thereby validating its generalizability and clinical utility.

	**Clinical**		**Radiomics**		**Combined**	
	OG	L-XGB	OG	L-XGB	OG	L-XGB
**Internal validation**						
Accuracy	75.38%	65.90%	83.85%	72.77%	72.30%	74.14%
Sensitivity	29.17%	57.32%	62.50%	63.41%	79.17%	69.51%
Specificity	85.85%	67.89%	88.68%	74.93%	70.75%	75.21%
**Prospective test**						
Accuracy	76.00%	61.00%	74.00%	71.00%	72.00%	73.00%
Sensitivity	43.48%	73.91%	82.61%	78.26%	70.13%	73.91%
Specificity	85.71%	57.14%	71.43%	68.83%	78.26%	72.73%
**External test**						
Accuracy	64.00%	58.00%	75.00%	71.00%	75.00%	74.00%
Sensitivity	50.00%	63.64%	72.73%	72.73%	75.64%	68.19%
Specificity	67.95%	56.41%	75.64%	70.51%	72.72%	75.64%

Supplementary Table S2. Gene Set Variation Analysis (GSVA) Pathway Correlations with LANTERN-XGB Risk Predictions. This table presents the significant associations between patient-specific GSVA enrichment scores (derived from MSigDB Hallmark gene sets) and the predicted probabilities of occult lymph node metastasis generated via Leave-One-Out (LOO) cross-validation. Pathway: The evaluated biological hallmark gene set. Correlation: The correlation coefficient evaluating the relationship between the pathway's enrichment score and the model's predicted OLM risk. Positive values indicate pathways that are upregulated in patients predicted to be at high risk (e.g., immune-inflammatory signaling, metabolic stress), whereas negative values indicate pathways that are downregulated in high-risk patients (e.g., tissue differentiation). P-value: The nominal statistical significance of the correlation. P-value adjusted: The adjusted p-value, corrected for multiple hypothesis testing. Only pathways demonstrating statistical significance (p-value adjusted<0.05) are reported.

**Pathway**	**Correlation**	**P-value**	**P-value adjusted**
HALLMARK_MYOGENESIS	-0.77	9.1E-05	3.3E-03
HALLMARK_UNFOLDED_PROTEIN_RESPONSE	0.69	1.0E-03	1.9E-02
HALLMARK_INTERFERON_GAMMA_RESPONSE	0.66	2.1E-03	2.6E-02
HALLMARK_INTERFERON_ALPHA_RESPONSE	0.63	3.5E-03	2.7E-02
HALLMARK_G2M_CHECKPOINT	0.62	4.0E-03	2.7E-02
HALLMARK_E2F_TARGETS	0.61	5.2E-03	2.7E-02
HALLMARK_UV_RESPONSE_DN	-0.60	5.8E-03	2.7E-02
HALLMARK_IL2_STAT5_SIGNALING	0.60	5.9E-03	2.7E-02
HALLMARK_MYC_TARGETS_V2	0.59	6.9E-03	2.8E-02
HALLMARK_MYC_TARGETS_V1	0.58	8.4E-03	3.0E-02
HALLMARK_ALLOGRAFT_REJECTION	0.57	1.0E-02	3.1E-02
HALLMARK_MITOTIC_SPINDLE	0.57	1.0E-02	3.1E-02
HALLMARK_DNA_REPAIR	0.55	1.3E-02	3.6E-02
HALLMARK_MTORC1_SIGNALING	0.53	1.8E-02	4.7E-02

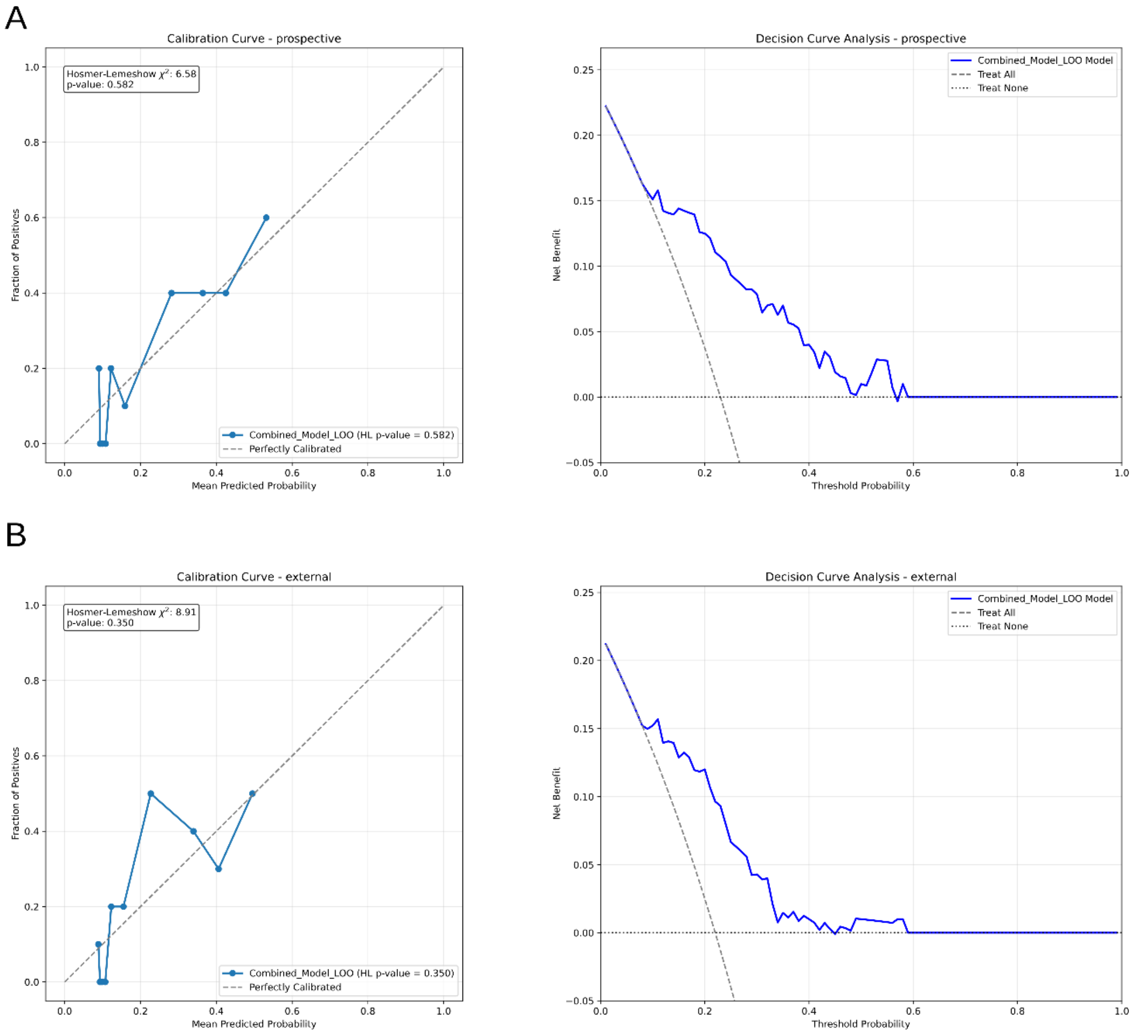

Supplementary Figure S1 Clinical Utility and Calibration of the LANTERN-XGB Model. The figure evaluates the real-world applicability and decision-making value of the multi-modal predictions in the prospective test cohort **(A)** and the independent external test cohort **(B)** for the combined clinical/radiomics model. **Left sub-panels:** Calibration curves assessing the agreement between the model-predicted probabilities of occult lymph node metastasis (x-axis) and the actual observed clinical frequencies (y-axis). The diagonal dashed line represents perfect algorithmic calibration. **Right sub-panels:** Decision Curve Analysis (DCA) evaluating the net clinical benefit (y-axis) of the LANTERN-XGB framework across a continuum of theoretical risk thresholds (x-axis). The model's performance is compared against the default clinical strategies of assuming all patients have occult metastasis ("treat all", descending line) or assuming no patients have metastasis ("treat none", horizontal baseline). Across both validation cohorts, the LANTERN-XGB pipeline demonstrates a superior net benefit over standard baseline strategies across a wide range of clinically relevant decision thresholds.
